# Comparative analyses imply that the enigmatic sigma factor 54 is a central controller of the bacterial exterior

**DOI:** 10.1186/1471-2164-12-385

**Published:** 2011-08-01

**Authors:** Christof Francke, Tom Groot Kormelink, Yanick Hagemeijer, Lex Overmars, Vincent Sluijter, Roy Moezelaar, Roland J Siezen

**Affiliations:** 1 TI Food and Nutrition, P.O.Box 557, 6700AN Wageningen, The Netherlands; 2 Kluyver Centre for Genomics of Industrial Fermentation, Julianalaan 67 2628 BC Delft, The Netherlands; 3 Wageningen University and Research Center, Laboratory of Microbiology, Dreijenplein 10, 6703 HB Wageningen, the Netherlands; 4 Netherlands Bioinformatics Centre, Geert Grooteplein 28 6525 GA Nijmegen, The Netherlands; 5 Wageningen University and Research Center, Food and Biobased Research, PO Box 17, 6700 AA The Netherlands; 6 Center for Molecular and Biomolecular Informatics (260), NCMLS, Radboud University Nijmegen Medical Center, P.O.Box 9101, 6500HB Nijmegen, The Netherlands

**Keywords:** biofilm, enhancer binding protein, exopolysaccharide, lipopolysaccharide, nitrogen assimilation, motility, peptidoglycan

## Abstract

**Background:**

Sigma-54 is a central regulator in many pathogenic bacteria and has been linked to a multitude of cellular processes like nitrogen assimilation and important functional traits such as motility, virulence, and biofilm formation. Until now it has remained obscure whether these phenomena and the control by Sigma-54 share an underlying theme.

**Results:**

We have uncovered the commonality by performing a range of comparative genome analyses. A) The presence of Sigma-54 and its associated activators was determined for all sequenced prokaryotes. We observed a phylum-dependent distribution that is suggestive of an evolutionary relationship between Sigma-54 and lipopolysaccharide and flagellar biosynthesis. B) All Sigma-54 activators were identified and annotated. The relation with phosphotransfer-mediated signaling (TCS and PTS) and the transport and assimilation of carboxylates and nitrogen containing metabolites was substantiated. C) The function annotations, that were represented within the genomic context of all genes encoding Sigma-54, its activators and its promoters, were analyzed for intra-phylum representation and inter-phylum conservation. Promoters were localized using a straightforward scoring strategy that was formulated to identify similar motifs. We found clear highly-represented and conserved genetic associations with genes that concern the transport and biosynthesis of the metabolic intermediates of exopolysaccharides, flagella, lipids, lipopolysaccharides, lipoproteins and peptidoglycan.

**Conclusion:**

Our analyses directly implicate Sigma-54 as a central player in the control over the processes that involve the physical interaction of an organism with its environment like in the colonization of a host (virulence) or the formation of biofilm.

## Background

Sigma factors specify bacterial transcription by binding to a characteristic promoter and thereby recruiting the associated RNA polymerase to that promoter. Ordinarily, the expression of genes/operons is controlled by the so-called 'housekeeping' sigma factor 70. However, most bacteria possess a larger repertoire of sigma factors of the Sigma-70 family, where each additional factor is associated with a specific programmed response [1]. For instance, in *Escherichia coli *and related Gamma-proteobacteria the entry into stationary phase and the adaptation to starvation is associated with Sigma-S [2,3], whereas the response to heat shock and similar stresses is mediated by Sigma-32 (e.g. [4,5]). In *Bacillus subtilis*, sporulation is orchestrated by 5 sigma factors (Sigma-E, F, G, H and K) [6], whereas the general stress response is controlled by Sigma-B [7,8]. In many species, particular extracellular signals are translated into an appropriate response by ECF sigma factors [9].

There is one sigma factor that seemingly does not fit in this picture as it has been associated with a range of physiological phenomena instead of with a singular response. Sigma-54 (gene *rpoN *in *E.coli*, *sigL *in *B. subtilis*) constitutes an evolutionary separate protein family and is found widely distributed among the bacterial kingdom, although there are phyla that lack the protein [10,11]. It binds to a characteristic -24/-12 promoter [12-14] and absolutely requires the input of free energy (ATP) from an associated activator to initiate transcription [15,16] (see [17,18] for recent reviews on the mechanism). In most cases the activator binds to an enhancer element located upstream of the promoter and hence is referred to as Enhancer Binding Protein (EBP^54^). The EBP^54^s bind the DNA as inactive dimers, but upon reception of the appropriate signal they assemble into oligomeric rings [19,20], with hexamers constituting the oligomeric active state [21]. A large variety of EBP^54^s exists and although some species possess one, for instance *Chlamydia trachomatis *[22] and *Lactobacillus plantarum *[23], most species have more variants. *B. subtilis *and *E. coli *were reported to have five (see [24]) and twelve [25], respectively, and *Myxococcus xanthus *to have fifty-three [26]. However, many of the reported numbers need correction (as described later) because the previous analyses have included EBP^54 ^paralogs that have lost the interaction with Sigma-54, like TyrR [27] and DhaR [28] in *E. coli *and HupR in *Rhodobacter capsulatus *[29,30].

Historically, Sigma-54 has been linked to the regulation of nitrogen metabolism. The protein was discovered as a positive regulatory factor needed for the expression of enterobacterial glutamine synthetase [31], before it was recognized that the protein is actually a sigma factor [32]. However, it was soon after established that Sigma-54 mediated control of transcription is not only connected to nitrogen assimilation but to a wider range of cellular processes and physiology in the enterobacteria [25,33]. Since then, it was shown that its role also encompasses the regulation of for example: flagellar biosynthesis in *E. coli *[34]; carboxylate uptake, central metabolism and flagellar biosynthesis in *Geobacter sulfurreducens *[35]; phosphotransferase system (PTS)-mediated carbohydrate uptake in the Gram-positive species *Lactobacillus plantarum *[23] and *Listeria monocytogenes *[36]; and PTS-mediated regulation in Gram-positive as well as Gram-negative organisms [37,38]; osmotolerance in *Listeria *[39]; the utilization of compounds like gamma-aminobutyrate in *Bacillus *[40], and the less familiar biphenyl in *Ralstonia metallidurans *[41] and toluene, xylene (see [42]) and choline [43] in *Pseudomonas*; Type III secretion system mediated pathogenicity in *Pseudomonas syringae *[44] and Type VI secretion system mediated toxin secretion in e.g. *Aeromonas *and *Marinomonas *[45]; the adaptation to cold shock in *B. subtilis *[24]; the control of Sigma-S [46], lipoprotein biosynthesis and virulence [47] in *Borrelia burgdorferi*; acid resistance of pathogenic *E. coli *O157 [48]; biofilm formation by *Burkholderia *[49]; and motility, biofilm formation, luminescence, and colonization in *Vibrio fischeri *[50,51]. The above plethora of associations has up to now obscured the definition of a general underlying functional theme that adds to the accepted associations with nitrogen metabolism and flagellar biosynthesis.

Several comparative studies have been performed for Sigma-54 and EBP^54^-mediated regulation [10,15,16,52], but no unifying biological theme was identified. An in-depth comparative analysis was made for *E. coli *by [25]. These authors concluded that nitrogen assimilation was one of the main processes connecting the Sigma-54 regulon. Besides, they found that a substantial fraction of the associated functions was seemingly unrelated. Some additional associations were proposed on basis of a comparative analysis on *Pseudomonas putida*, including links to carbon metabolism and flagellar biosynthesis [53]. Since the last comprehensive comparative study in 2003 a considerable number of genomes has been sequenced, allowing us to make a new overview of the presence of Sigma-54 and the EBP-activators. Surprisingly, we found a clear-cut connection between the presence of the system and characteristic morphological features. To enhance the identification of true EBP^54 ^activators and Sigma-54 promoters, we have tested and employed a straightforward motif search algorithm that directly relates to sequence similarity. Redefinition of the -24/-12 promoter and the similar motif search (SMS) approach allowed for the reliable identification of promoter sites in all species. Finally, we have analyzed the function annotations that were highly represented (intra-phylum) and conserved (inter-phylum) within the genomic context of all genes encoding Sigma-54, its activators and its promoters, to identify common functional traits.

Conserved genome context, i.e. synteny, is a strong indicator of a functional relationship between genes [54,55] and it is therefore being used broadly to guide function prediction. In principle, the fact that encoded functions that show a conserved genomic proximity are mostly related does not only hold for genes, but by necessity extends in the direction of genetic (regulatory) elements [56], and thereby also in the direction of associated regulators (see e.g. [57]) and their (in)activating signals [58]. As a consequence, a comparative analysis of the conserved genome context of regulators and regulatory elements should yield clues regarding the particular associated stimuli and responses. Although regulatory routes can vary between species much more than metabolic pathways, the functional associations at a higher hierarchical level (i.e. in terms of process, response and/or physiology) are far less variable. For instance, the bacterial PTS mediates the transport and phosphorylation of carbohydrates by means of phosphoenolpyruvate via the same phosphotransfer mechanism in all species and, at a higher hierarchical level, the system controls the same processes like catabolite repression and chemotaxis [37,59]. Nevertheless, the precise regulatory interactions of the PTS and the intracellular signals that connect the organism's physiological state to the metabolic level differ significantly between groups of species (i.e. catabolite repression involves EIIA^Glc ^and cAMP in *E. coli*, whereas it involves HPr and Fructose-1,6-bisphosphate in *B. subtilis*). The above implies that underlying functional themes that can not be discovered directly, for instance by studying conserved gene-associations of a particular regulator, may be discovered by mapping the associated functions at a higher hierarchical level (like pathways).

Absolute conservation will be relatively rare because of the earlier noted variability in the specific regulatory associations. To take such variability into account, we included in our analysis those functional associations that are highly represented within a phylum/class but are at the same time evolutionary conserved, that is present within several phyla/classes. Associations that fulfill this criterion can be viewed as cross-phylum (or cross-class) conserved function tendencies. By mapping of the conserved annotations present in the genetic context of the genes encoding Sigma-54, its EBP^54^-activators and its promoters, we discovered that there is indeed a common functional theme related to Sigma-54-mediated regulation, namely, the control of the transport and biosynthesis of the molecules that constitute the bacterial exterior, which encompass the extracellular polysaccharides (EPS), flagella, lipopolysaccharides (LPS), lipoproteins and the building blocks of the peptidoglycan cell wall.

## Results

### Taxonomic diversity of Sigma-54 and its Enhancers

Completely sequenced and published prokaryotic genomes listed in the GOLD database [60] were searched at the protein level for homologs of Sigma-54 and the related enhancer-binding proteins (EBP^54^s). Experimentally characterized proteins were used to seed the search (see methods). In the case of Sigma-54, the sequences of the *E. coli *and *B. subtilis *proteins sufficed to recover all orthologs using a low cutoff value (1e^-20^) (hits given in additional file 1). In the case of the far more diverse EBP^54^s, a list of 34 experimentally verified proteins from diverse species was compiled and it required extensive filtering to reduce the initial list of putative homologs (see methods and below). The final list is given in additional file 2 and corresponds well with earlier analyses performed at a smaller scale [16,52].

The collection of sequences exhibited a near perfect match between the presence in a genome of Sigma-54 and its EBP^54^-activators. A well-known exception was formed by the Neisseria strains that have a gene encoding Sigma-54 but lack an obvious candidate EBP^54^. However, the *Neisserial *Sigma-54 protein is inactive because the two helices that interact with the -13 promoter region [61] are missing in this protein [62]. The other exceptions were *Borrelia hermsii *DAH, *Borrelia turicatae *91E135, *Fusobacterium nucleatum nucleatum *ATCC 25586 and *Jannaschia *sp. CCS1, which possess a putative EBP^54 ^but lack a Sigma-54 ortholog. As the initial search was performed using the translated protein sequences we also searched the nucleotide sequence directly and found that the chromosome of *B. hermsii *carries an intact *rpoN *gene that was obviously missed in the original annotation procedure. However, we could not identify *rpoN *in *B. turicatae*. A rationale for the absence of Sigma-54 in *Fusobacterium nucleatum nucleatum *ATCC 25586 and *Jannaschia *sp. CCS1 will be given in the next section.

**Figure 1:**
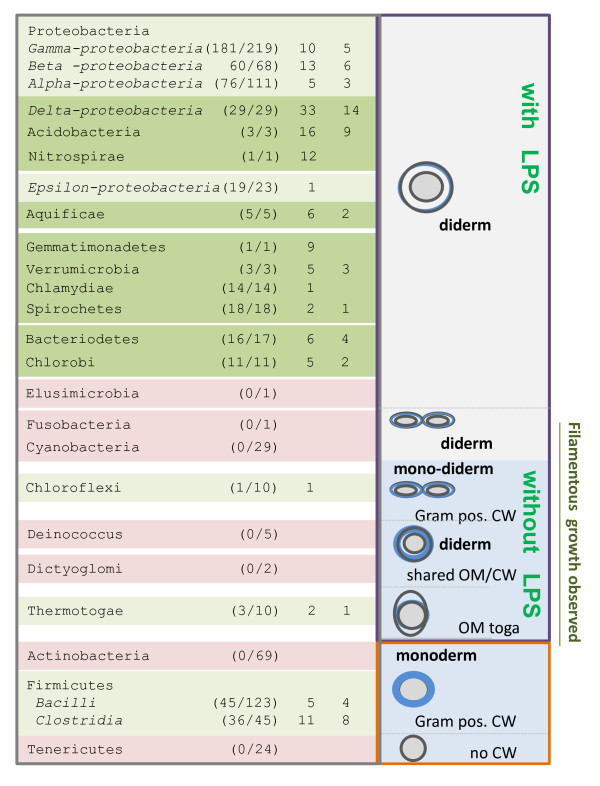
**Taxonomic distribution of Sigma 54 and the associated Enhancer Binding Proteins**. The phyla Proteobacteria and Firmicutes have been divided into the constituent classes. Between brackets, the number of genomes with Sigma-54 over the total number of sequenced genomes is listed followed by the average number of EBP^54^s with a discrete standard deviation. The corresponding data can be found in additional file 1. The ordering of the phyla is based on conserved gene arrangement comparisons [162], a concatenated alignment of 22 single-copy conserved genes [163] and the analysis of conserved indels [164]. Gemmatimonas was placed according to [165], Nitrospira according to [166] and Thermatogae according to [167]. On the right, the cell morphology in terms of number of membranes (monoderm vs. diderm), presence of LPS (from [64]) and nature of the cell wall peptidoglycan (Gram-, Gram+ or other) is given. The majority of phyla represent diderms, except for Tenericutes, Firmicutes and Actinobacteria. Chloroflexi are probably also monoderm [168] and some have been shown to have a thick cell wall and stain Gram positive [169]. *Deinococcus radiodurans *has a complex Gram + like cell wall that includes outer membrane-like structure and the cell wall and outer membrane can be shared by multiple cells [170]. *Dictyoglomus thermophilum *is diderm but can grow in bundles or spherical bodies which are surrounded by a common outer membrane [171]. Finally, the Thermotoga have an outer sheath-like envelope ('toga') and an atypical thin cell wall [172].

Sigma-54 and EBP^54^s were found in the majority of sequenced species (522 of 842 evaluated genomes) and in the majority of the evaluated phyla, as illustrated in Figure 1. Remarkably, their presence is almost absolutely conserved in species that are diderm (i.e. that have two cellular membranes [63]) and have an outer membrane that mainly consists of lipopolysaccharides (LPS) [64]. The diderm species in which Sigma-54 and its activators are absent represent mostly endosymbionts (> 90%), e.g. species from the Genus Bartonella, Buchnera, Ehrlichia, Francisella, Neisseria and Rickettsia, and *Elusimicrobium minutum *[65]. Furthermore, the proteins are also absent from all 29 sequenced species/strains of the phylum Cyanobacteria. For the monoderm phyla and for the diderm phyla that have different LPS or lack LPS altogether the picture is inverse, i.e. in most of the related species Sigma-54 and EBP^54^s are absent. They were identified only in some Chloroflexi and Thermotogae. In contrast, they are present in many Firmicutes, that is in most species of the class Clostridia and in the sporulating species of the class Bacilli. In addition, they were found in Listeria, *Enterococcus faecalis *and in the closely related Lactobacilli *Lactobacillus plantarum*, *Pediococcus pentosaceus *and *Lactobacillus casei *(only strain BL23).

**Figure 2:**
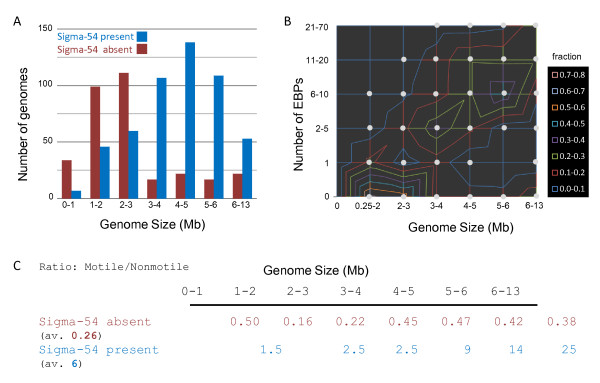
**Distribution of genome size (A), the number of EBP^54^-activators (B), and motility (C), for species with (blue) and without (red-brown) Sigma-54**. A) the analyzed species were binned according to genome size in bins of one Mbase, and divided in two groups that related to the presence or absence of Sigma-54. B) for every size-bin the fraction of genomes with a particular number of EBP-activators was determined and a height-plot was created. The grey dots indicate the data points. The contour was generated with Microsoft Office Excel 2007. C) Within every bin the fraction of motile species was determined for the genomes with Sigma-54 and without. The corresponding data can be found in additional file 1.

There was an overall correlation between the size of the genome and the presence of Sigma-54 as illustrated in Figure 2A. Most endosymbionts or facultative intracellular species have a small genome (< 1.5Mb) and lack Sigma-54, although some (i.e. Borrelia and Chlamydia) do have Sigma-54 and contain one activator. In addition, species of the phyla Actinobacteria and Cyanobacteria have much larger genomes (up to 9 Mb) but lack the Sigma-54 system. A small number of Proteobacteria, mostly species/strains from the orders Burkholderiales, Rhizobiales and Xanthomonadales (see also [10,66-68]), possess two variants of the sigma factor and the sequenced *Rhodobacter sphaeroides *strains even contain three or four variants [69,70]. In the case that Sigma-54 is present, there is a strong positive correlation between the number of encoded EBP^54^s and the size of the genome as might be expected (see Figure 2B). On top of that, there appeared to be a clear phylum/class dependency ranging from the Delta-proteobacteria with around 33 EBP^54^s on average to the Epsilon-proteobacteria, Chlamydia and Spirochetes with on average 1 or 2 activators (Figure 1). Importantly, a strong positive correlation between reported motility and the presence of Sigma-54 was observed and this correlation appeared predominantly independent of genome size (Figure 2C). The strong correlation fortifies the general view that one of the common functional themes of Sigma-54 mediated control is the association with the synthesis of flagella and or pili, an association that has been observed for many species (reviewed in [71]). Other clear correlations with particular bacterial lifestyles (as represented in the GOLD database) were not observed.

### Sequence-based identification and characterization of the EBP^54^s

**Figure 3:**
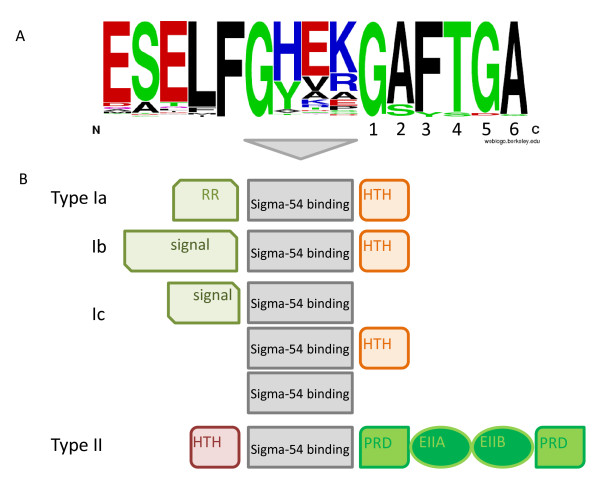
**Sequence composition of the Sigma-54 Enhancer Binding Proteins**. A) The 'GAFTGA' sequence logo of the 4970 putative functional Sigma-54 related EBPs. Data from literature and similarity in chemical structure were used to categorize the substitutions into those that relate to functional EBP^54^s, those that will probably relate to functional EBP^54^s, and those that will abolish the interaction with Sigma-54. The effect of amino acid substitutions on the EBPs capacity to activate Sigma-54 mediated transcription has been studied by [75,76]. Furthermore, some experimentally validated activators carry specific substitutions: G_1 _is replaced by N in the only EBP^54 ^of *Paracoccus denitrificans *and *Ruegeria pomeroyi *(putative: ADEHS); A_2 _is replaced by S in LevR of the Bacilli (putative: TGIVMC; inactive: DN); F_3 _is replaced by Y in TouR of *Pseudomonas stutzeri *(other replacements inactive); T_4 _is replaced by S in BkdR of *B. subtilis *and by E in PhhR of *Pseudomonas aeruginosa *(putative: D; other replacements inactive); G_5 _is replaced by D in FlgR, the only EBP^54 ^of Campylobacter and other Epsilon-proteobacteria (putative; EAHNS); and A_6 _is replaced by S in PrpR of *E. coli *(putative: TGIVMC; inactive DN). B) Schematic representation of the four basic architectures of functional EBP^54^s. The types were distinguished on basis of their domain organization: Ia) N-terminal signal recognition domain of the response regulator (RR) type, followed by the central activator domain and a C-terminal DNA-binding domain of the HTH_8 PFAM family; Ib) different N-terminal signal recognition domain(s), followed by the central activator domain and a C-terminal DNA-binding domain of the HTH_8 PFAM family; Ic) an activator domain, but lacking the signal recognition domain (e.g. PspF, HrpRS, LafK) or the DNA-binding domain (e.g. CtcC, FlgR) or both (FleT); and II) N-terminal DNA-binding domain of the NtrC family, the central domain, and four phosphorylatable domains related to the PTS.

The list of EBP^54^s that was obtained in a BLAST search using the Sigma-54 interaction/activator domain (PFAM: PF00158), was initially filtered using two criteria based on cut off value (see methods). In this way, 5494 potential EBP^54^s were identified. The list contained many false positives (~10%), which were mostly proteases and Mg^2+ ^chelatases [72] as these are the closest relatives of the Sigma-54 interaction/activator domain [73]. To remove false positives, we analyzed the presence of the characteristic 'GAFTGA' amino acid sequence that is essential for the interaction between the activator and the sigma factor [74]. It has been established that single residue changes within the sequence element and especially within the central phenylalanine and threonine, reduce the transcriptional activity considerably and mostly abolish it [75,76]. Given the reported constraints, which are summarized in the legend of Figure 3, 4850 true and 121 putative EBP^54^s were obtained (additional file 2). The 'GAFTGA' sequence within this set is highly conserved (see Figure 3A), and the importance of this conservation is corroborated by the substitutions/deletions that lead to 'non-functional' EBP^54 ^homologs, like in TyrR, DhaR of *E. coli *and HupR of *R. capsulatus*, which have been shown to be Sigma-70 dependent ([27,28] and [29,30], respectively). Similar changes were observed within the protein sequence of the VpsR regulator of polysaccharide production in *Vibrio cholerae *and the YplP regulator of the cold shock response in *B. subtilis*. These proteins were proposed to be Sigma-54 dependent ([77] and [24], respectively), but considering the lack of compelling experimental evidence and the clear deletion within the 'GAFTGA' sequence (see additional file 3) they are probably not. We found that around 110 of the recovered homologs showed such minor deviations and within this group there appeared to be a preference for the substitution of the Thr/Ser at position 4 by a Pro (additional file 3). In the putative EBPs of *Fusobacterium nucleatum nucleatum *ATCC 25586 and *Jannaschia *sp. CCS1, Thr/Ser at position 4 is replaced by Glu. Considering the fact that both organisms lack Sigma-54, the observed replacement may have resulted in a Sigma-70 dependency, like observed for DhaR and TyrR. The fact that Fusobacterial EBP is orthologous to PhhR of *Pseudomonas aeruginosa*, a paralog of TyrR which was shown to be able to replace TyrR as a repressor of the *aroF-tyrA *operon in *E. coli *[78], supports this assumption.

Previous comparative analyses of the Sigma-54 associated EBP^54^s made clear that the Sigma-54 activators connect to a wide spectrum of input signals [15,52]. In fact, this could be one of the main reasons that a common functional theme thus far has remained obscured. Currently, the PFAM database describes around 136 different domain compositions (architectures) for EBP^54^s. Nevertheless, some generalizations can be made. All EBP^54^s possess a central activator domain, which is responsible for the interaction with Sigma-54 and provides the ATPase activity that is required to initiate transcription. In addition, most enhancer-binding proteins have one to several signal binding/recognition domains and a DNA-binding domain, although some EBP^54^s lack either the former or the latter [18,44,79,80]. Basically two main domain organizations can be discerned, which can be further subdivided according to specific domain composition as indicated schematically in Figure 3B.

To specify the functional associations of the Sigma-54 activators, PFAM domain annotations were collected from the Uniprot database and used to make an inventory of the specific EBP^54 ^categories. We found that around 43% of the activators were of type Ia, thus related to the transduction of extracellular signals/cues via a two-component system histidine kinase (for review on TCSs see [81,82]). Furthermore, around 12% of the activators contained a PAS domain and around 10% a GAF domain (both type Ib). Earlier comparative analyses have shown that these two are the most wide-spread domains among bacterial signaling systems [83,84]. The PAS domain has been linked to a variety of (small) signaling molecules [85] and the same holds for the GAF domain (see [38]), although it was originally linked to the recognition of cyclic nucleotides (see [86]). In several cases the PAS and GAF domain occurred in conjunction with another signal-recognition domain, which is suggestive of dual activation and or signal integration. Around 4% of the activators was of type Ic, lacking a DNA-binding domain and/or signal recognition domain. Finally, around 3% of the activators was directly linked to signaling via the PTS. These activators included proteins of type Ib with an HPr signal-domain found in Clostridia (0.3%) [87] and the proteins constituting type II (2.5%), with four different phosphorylatable domains (2xEII and 2xPRD; see [37]). Notably, a substantial number of EBP^54^s lacked a clear PFAM annotation of the putative signal recognition domain. Given the specificity of the Hidden Markov Models used to identify the response regulator domain one may assume that these activators mainly represented types 1b and 1c. Around 8% of all EBP^54 ^sequences was shorter than 400 amino acids, making them likely of type 1c. Based on the above, type 1b activators represented around 46% of the EBP^54^s identified.

More than half of the activators (~60%) could be annotated in a coarse way, on basis of the similarity to experimentally verified EBP^54^s. As expected, the coverage within the Gamma-proteobacteria and the Firmicutes was higher than for other classes. Nevertheless, the data clearly indicate that the 'membership' and cross-phylum conservation is much higher for certain annotations than for others, as illustrated in Table 1. More explicitly, the annotations within the largest group include connections to the catabolism of short-chain fatty-acids and chemotaxis (AtoC), the synthesis of lipoprotein (Rrp2) and the response to cyclic-di-GMP (e.g. ZraR), a second messenger that regulates cell-surface-associated traits [88,89]. Other highly represented and cross-phylum conserved associations include the control of nitrogenase (VnfA, 11 phyla/classes), nitric oxide reduction (NorR), nitrogen assimilation and the production of EPS and LPS (NtrC), the transport and metabolism of (di-)carboxylates (AcoR, DctD, FhlA, GabR, PrpR), flagellar synthesis (AdnA, FleQ, FleR, FlrA, FlrC) and the degradation and uptake of various kinds of cell wall (poly-)saccharides (CelR- and LevR-like). Minor activities not listed include the sensing, transport and metabolism of hydrocarbons (XylR-like [42]), aromatic amino acids (PhhR [78] or CbrB [90]) and the production of the EPS alginate (AlgB [91]).

**Table 1:** Main classes of Sigma-54 related activators and the connected functional data.

	annotation	nr	% of total	phyla/ class	members	associated processes
Ia)	AtoC-like	408	8.2	16	AtoC, FrgC, HydG, Rrp2, ZraR	AtoC: catabolism of short chain fatty acids induced by **acetoacetate**; biosynthesis of **polyhydroxybutyrate**; related to motility and expression of **flagella**r genes [124]; FrgC: developmental association in *Myxococcus xanthus *[180]; Rrp2: synthesis of **lipoproteins **in Borrelia [181]; ZraR: responds to **cyclic-di-GMP **[182]
	NtrC-like	302	6.1	5	NtrC, NRI	**nitrogen assimilation **in Gamma-proteobacteria (see [25]); **choline catabolism **in *Pseudomonas aeruginosa *[43]; biosynthesis of **EPS **and **LPS **in *V. vulnificus *[183]and *P. aeruginosa *[184];
	DctD-like	209	4.2	3	DctD	(C4) **dicarboxylate **transport, associated with symbiosis in Rhizobia [137]
	QseF-like	117	2.4	3	QseF, YfhJ	production OM protein in response to host **pheromone **or **sulphate **and **phosphate **stress [185]
	FleR-like	86	1.7	4	FleR, FlrC	**motility **and adhesion to mucin in *Pseudomonas aeruginosa *[186]

Ib)	AcoR-like	216	4.3	6	AcoR	**acetoin **metabolism in *B. subtilis *[187]
	NorR-like	146	2.9	3	NorR	**nitric oxide **detoxification in *E. coli *[188]
	PrpR-like	129	2.6	3	PrpR	control of **propionate **metabolism in *Salmonella *[189]
	FhlA-like	91	1.8	4	FhlA, HyfR	FhlA: controls expression of formate hydrogen lyase and is induced by **formate **[190]
	GabR-like	90	1.8	4	GabR	GabR: control of the **gamma amino-butyric acid **shunt in *Bacillus thuringiensis *[40]
	FleQ-like	81	1.6	1	FleQ, AdnA, FlrA	synthesis of **flagella **in *Legionella pneumophila *[115]
	VnfA	51	1.0	11	VnfA	control of **nitrogenase **expression (see [191])

Ic)*	PspF	139	2.8	3	PspF*	control of **membrane stress **response (see [80])

II	LevR-like	123	2.5	3	CelR, LevR	LevR: controls *lev *operon in *B. subtilis*. Operon includes PTS transport of **polyols **and other **sugar derivatives **[130]; CelR: controls *cel *operon in *Geobacillus stearothermophilus*. Operon includes PTS with strong activity towards plant **cell wall carbohydrates **[131]; EsuR: controls esu operon, which is related to **acetyl-sugar **uptake and hydrolysis [132] lmo1721: *cel *operon in *Listeria monocytogenes*; control of **virulence **genes [133]

Column one lists the type of EBP^54 ^and column two a general group annotation. The annotation was based on BLAST scores against a list of 60 experimentally characterized activators (given in additional file 3) and represents around 60% of the identified activators. Columns 3 and 4 provide the (relative) number of activators that are in the group and column 5 gives the number of phyla/classes that are represented (maximum 17). Column 6 lists the characterized activators that are member the group. The final column provides a function description for the specified group member as retrieved from literature, where the main metabolite or process is in bold type.*The EBPs of type Ic, include PspF [80], HrpR (0.3%) [44,176] and LafK (0.3%) [177] which lack a separate signal recognition domain, Chlamydia CtcC [178] and Helicobacter FlgR [179] which lack a DNA-binding domain. and *R. sphaeroides *FleT [117] which lacks both domains.

### Identification of Sigma-54 promoters

**Figure 4:**
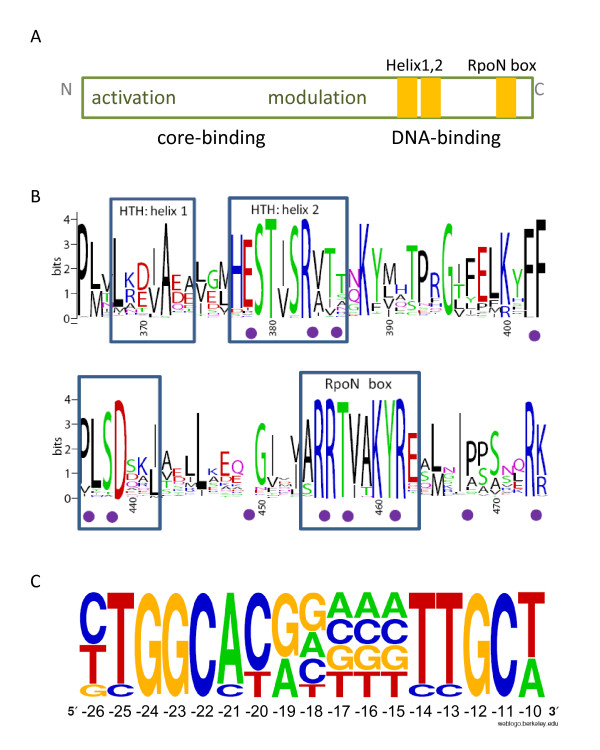
**Sequence features of Sigma-54 and its promoter**. A) Functional architecture of the Sigma-54 sequence (adapted from [10]). The first HTH is responsible for recognition of the -12 element as was demonstrated by [61]. The solution structure of the C-terminal domain of *Aquifex aeolicus *Sigma-54 bound to the promoter implied that the RpoN box [173] and two flanking stretches interact directly with the -24 element of the promoter [174], confirming an earlier assertion of [175]. B) Sequence logo of the two HTH elements as present in all analyzed Sigma-54 proteins. The residue pairs whose substitution abolished binding activity in the elaborate Ala-Cys scanning mutagenesis study by [93], are marked by purple dots in-between. C) Reduced promoter sequence motif. The motif is based on the 85 promoters with validated transcription start site as collected by [14]. The position relative to the transcription start is given on the x-axis.

The Sigma-54 amino acid sequence can be divided into three distinct parts that show a high degree of conservation [92] as depicted in Figure 4A. However, the sequences that link the parts can vary considerably depending on the species. The C-terminus contains two separate Helix-Turn-Helix motifs (HTH) that are responsible for recognition of the -24 and -12 promoter elements, respectively. The multiple sequence alignment of all recovered Sigma-54 sequences shows that the amino acid residues of the two HTH motifs are especially well-conserved (Figure 4B). The degree of conservation of the HTH residues corresponds remarkably well to the negative effect on activity that was measured after the substitution of residue pairs [93]. Considering the high degree of conservation within the DNA-binding sequence of Sigma-54, one would expect a similar degree of conservation for the promoter sequence. Indeed, a consensus promoter sequence has been defined earlier by combining experimentally verified promoters from a variety of species [14]. The consensus has been used by many researchers to search for new putative Sigma-54 binding sites (e.g. [94]). Nevertheless, it is very clear from the conservation pattern within the consensus promoter that some positions are not very informative. Therefore, we reduced the size of the promoter motif and smoothed those frequencies that appeared to be rather random, to arrive at a frequency representation of the Sigma-54 promoter as given in Figure 4C.

**Figure 5:**
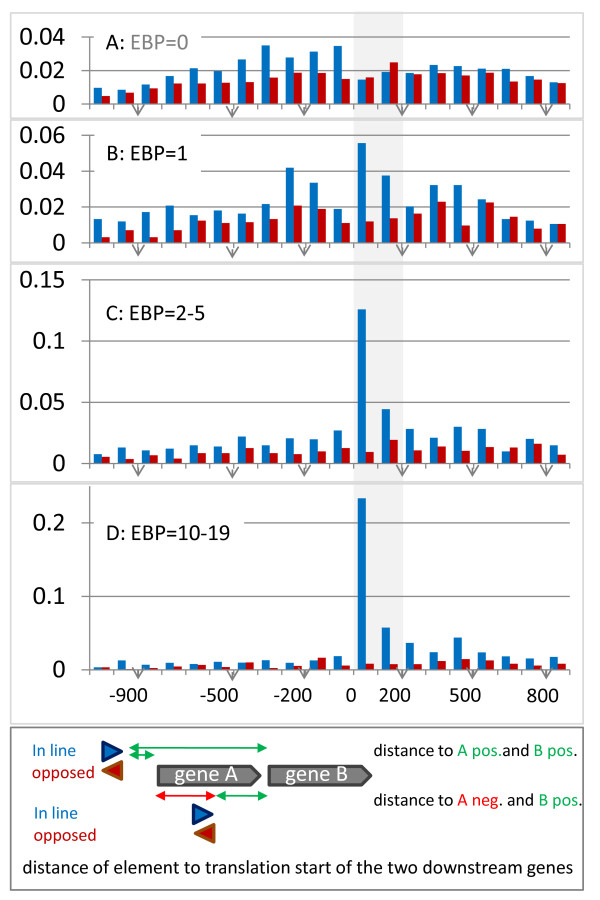
**Distribution of the genomic distance between the downstream genes and the sequence elements that are most similar to the Sigma-54 promoter motif**. The distance distribution (in bins of 100 nucleotides) was summed for A) all genomes that lack Sigma-54 and its activator (A; EBP = 0), and for those genomes that have Sigma-54 and one (B; EBP = 1) or multiple EBP^54^s (C; EBP = 2-5, D; EBP = 10-19). The distance distribution for genomes with EBP = 6-9 and EBP ≥ 20 are similar to the latter and therefore not shown. For every identified element two distances were included as indicated in the figure inset. As a result the distribution actually represents the sum of two distributions. The distance was taken from the -11 position of the promoter to the predicted translation start of the gene (situation i). In case the element was located within a gene (situation ii) the distance to the first gene was taken as negative. In blue the distance distribution is given for the cases that the gene downstream is oriented in line with the predicted promoter and in red for the cases that it opposes the promoter. The sum of the distributions was normalized.

The reduced Sigma-54 promoter motif was used to identify similar sites in all the studied genomes. To that end, we formulated a straightforward frequency-based scheme to score similarity and implemented the scheme in a similar motif search (SMS) tool (see methods). SMS was tested to predict the well-studied CcpA and Spo0A regulon in *B. subtilis *and the simple scoring appeared as effective as MAST and more effective than HMMs in finding members of the respective regulons (see methods). The results of the similar motif search for the Sigma-54 promoter were evaluated by setting an arbitrary initial score threshold (85% of the maximum obtainable score), and then counting the number of occurrences in every genome. As expected, there was a clear correlation between the number of identified similar sites and genome size. In contrast, there was no clear difference between the number of potential binding-sites in organisms that have Sigma-54 *versus *organisms that do not. However, in case the orientation and the distance of the potential promoters with respect to the predicted translation start sites of the genes located downstream was taken in to account, the difference was obvious (as depicted in Figure 5). For organisms that possess Sigma-54, a large fraction of the most similar binding-sites is located in the region between 0 and 200 nucleotides upstream of a translation start (see Figures 5BCD), whereas in organisms that lack Sigma-54 the distribution of sites shows a slight decrease in this region (Figure 5A). In fact, this finding implies that those potential sites that are appropriately located to function as a promoter are highly likely to function as a genuine Sigma-54 promoter. Therefore, the list of potential sites could be reduced using a simple distance criterion (i.e. -50 - 300 nucleotides upstream of translation start) to yield a list of putative Sigma-54 controlled genes/operons (the results of the promoter identification are available at http://www.cmbi.ru.nl/bamics/supplementary/Franckeetal_2011_Sigma54theme).

The validity of the above identifications was substantiated by a comparison of several predicted species-specific Sigma-54 regulons with those reported in literature. The comparison included *B. subtilis *[95], *E. coli *[25,96], *Lactobacillus plantarum *[23] and *Pseudomonas putida *[53] (see additional file 4). All regulons that were compared showed a very good agreement for the high ranking predicted promoters (i.e. having > 85% of the maximum attainable score). The high-ranked predicted promoters captured more than 95% of the established sites in *Pseudomonas putida*, *B. subtilis *and *Lactobacillus plantarum*. In *E. coli*, 90% of the established Sigma-54 promoters was captured in case a slightly lower threshold was used (i.e. > 80% of maximum). The relatively higher number of less similar 'true' sites in *E. coli *is in line with earlier findings [25,34,96]. Not only did we observe excellent agreement between the predictions and established Sigma-54 promoters, we also identified new likely 'regulon' members. These include for instance: the genes lp_0562 (*nagA*, encoding an N-acetyl-glucosamine-6-phosphate deacetylase) and lp_0586 (*pts10A*, encoding a mannose PTS EIIA) in *Lactobacillus plantarum *WCFS1; the genes PP_0662 (encoding a threonine synthase), PP_4359 (*fliL*, encoding a flagellar basal body-associated protein) and PP_1705 (*nirB*, encoding the large subunit of nitrite reductase) in *Pseudomonas putida*; and the genes b3529 (*yhjK*, encoding a putative diguanylate cyclase, b1786 (*yeaJ*, encoding a diguanylate cyclase involved in the regulation of motility [97]), b2870 (ygeW, encoding an aspartate/ornithine carbamoyltransferase [98] and b4444 (*glmY*, encoding the small RNA that is involved in the activation of expression of glucosamine-6-phosphate synthase [99]) in *E.coli*. Expression of the small RNA encoding gene *glmY *was indeed proven to be Sigma-54 dependent in *E. coli *and other enterobacteria such as *Yersinia pseudotuberculosis *and *Salmonella typhimurium *[99,100].

### Conserved function tendencies in genomic context

We collected various kinds of annotations to identify a potential underlying functional theme, for genes: i) that share genome context with the gene encoding Sigma-54 (10 genes upstream and downstream); ii) that occur in transcriptional units containing an EBP^54^-activator encoding gene (operons and divergons); and iii) that are in transcriptional units preceded by a putative Sigma-54 promoter (see methods for more details). The annotations that were extracted from the reference databases included: COG (av. 73% ± 1%) and GO categories (63% ± 5%), PFAM (83% ± 3%) and Interpro (77% ± 1%) domains, KEGG orthologs (59% ± 5%), EC numbers (9% ± 2%), trivial gene names (22% ± 3%) and detailed function descriptions (67% ± 6%). The average coverage of the various annotations that is given between brackets for the three context collections shows that only part of the recovered genes was connected to annotation information. The COG categories, PFAM domains and KEGG orthologs together connect more than 80% of the complete set of genes to a function annotation and therefore these annotations should represent the overall genetically associated functions to a reasonable extent. The annotations were lumped in a phylum-specific manner and those annotations that were highly represented within a phylum or class (top 10 or 20, depending on number of genomes) and represented within several phyla/classes (≥ 2), were extracted. The results of the procedure can be found in additional files 5, 6 and 7, respectively, and are summarized in the following.

Table 2 presents an overview of the COG functional categories that were found over-represented in the set of genes that are directly (i.e. genomically) associated with the genes encoding Sigma-54 and its activators and with the identified Sigma-54 promoters. The representation was determined relative to the complete set of proteins in the COG database. There appeared to be six prevalent categories, namely: Energy production and conversion, Cell wall/membrane/envelope biogenesis, cell motility, post-translational modification, signal transduction and intracellular trafficking/secretion. Interestingly, the category 'amino acid transport' and biosynthesis was not over-represented.

**Table 2:** Representation of the COG categories within the genetic context of the genes encoding Sigma-54 and its EBP^54^s and of the promoters.

code	Description of category	**s54, EBP, prom**
A	RNA processing and modification	- - -
C	**Energy production and conversion**	- + +
D	Cell cycle control, cell division, chromosome partitioning	- - -
E	Amino acid transport and metabolism	- - +
F	Nucleotide transport and metabolism	- - -
G	Carbohydrate transport and metabolism	+ - -
H	Coenzyme transport and metabolism	- - -
I	Lipid transport and metabolism	- + -
J	Translation, ribosomal structure and biogenesis	+ - -
K	Transcription	- - -
L	Replication, recombination and repair	+ - -
M	**Cell wall/membrane/envelope biogenesis**	+ + +
N	**Cell motility**	- + +
O	**Posttranslational modification, protein turnover, chaperones**	+ + +
P	Inorganic ion transport and metabolism	- - -
Q	Secondary metabolites biosynthesis, transport and catabolism	- - -
T	**Signal transduction mechanisms**	+ + +
U	**Intracellular trafficking, secretion, and vesicular transport**	+ + +
V	Defense mechanisms	- - -

Over-representation of a particular COG category (with respect to the reference; http://www.ncbi.nlm.nih.gov/COG/old/) within at least half of the analyzed phyla/classes is indicated by +. The categories that were over-represented in more than two genetic contexts are indicated in bold. The functional categories that are not relevant because they are either non-specific (R, S) or mostly specific for eukaryotes (B, W, Y, Z) are not shown.

We then inspected the recovered annotations more specifically and found that various genes/function descriptions are often genomically associated to Sigma-54 mediated control. Not surprisingly, the main function associations found within the genetic context of the EBP^54^s and the best ranked promoters were very similar to those that have been reported in literature for the various Sigma-54 EBP-activators. The retrieved genes encoded: various histidine kinases, transport systems of metal ions, nitrate or ammonium, carboxylates, amino acids and cell wall (amino-)sugars and many flagellar proteins (*flg*, *fli*, *flh *operons). The genetic context of the Sigma-54 encoding gene was remarkably conserved throughout. In the didermal species it contained: *yhbJ*, encoding a regulator of glucosamine-6-phosphate synthase [101], *lptABC*, encoding the system responsible for the transport of LPS from inner- to outer-membrane [102-104], *kdsCD*, encoding genes related to the synthesis of the LPS building block 2-keto-3-deoxyoctanate (kdo) [105], PTS^Ntr^, a system implicated in the integrative regulation of nitrogen and carbon metabolism [37,38], and *yhbH *(HPF), a gene implicated in the phenomenon of ribosome hibernation that occurs in stationary phase [106]. In the Sigma-54 containing monoderm species (i.e. the Firmicutes), a conserved association was found with *yhbJ*, like in the diderm species, and with the genes encoding the central glycolytic enzymes and their regulator CggR [107].

**Figure 6:**
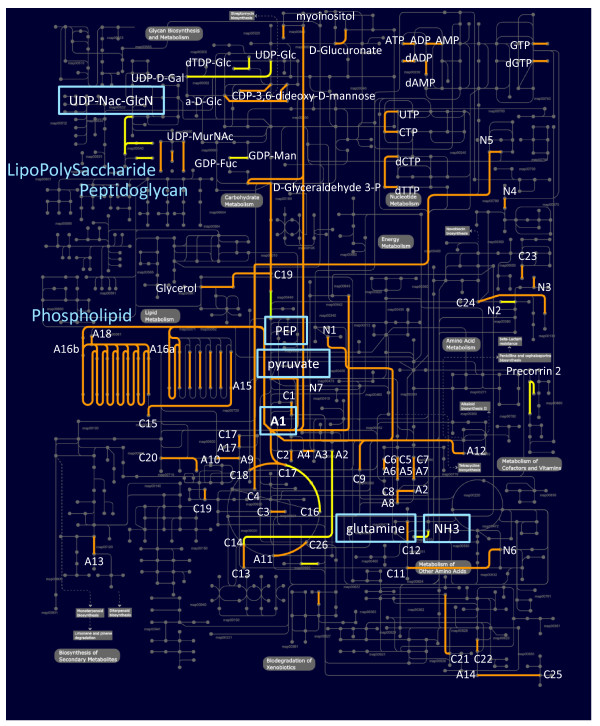
**Conserved function tendencies within the gene-associations of Sigma-54, its EBP^54^s and the Sigma-54 promoter**. The highly represented and cross-phylum conserved metabolic reactions were mapped using iPATH [108]. The reactions that relate to only Firmicutes are colored green, those that relate to diderm organisms only are colored yellow and those reactions represented in both monoderm and diderm species are given in orange. The routes associated with phospholipid, peptidoglycan and lipopolysaccharide biosynthesis are indicated and the related precursors are given in blue boxes. The metabolites that are associated to the recovered reactions fall in 3 distinct categories. i) CoA-related: A1, acetyl-CoA; A2, propanoyl-CoA; A3, propenoyl-CoA; A4, 3-hydroxypropanoyl-CoA; A5, 2-methylpropanoyl-CoA; A6, 3-methylbutanoyl-CoA; A7, 2-methylbutanoyl-CoA; A8, (R)-2-methyl-3-oxopropanoyl-CoA; A9, 2-butenoyl-CoA; A10, (S)-3-hydroxybutanoyl-CoA; A11, succinyl-CoA; A12, glutaryl-CoA; A13, 3alpha,7alpha-dihydroxy-5beta-cholestanoyl-CoA; A14, 3-oxoadipyl-CoA; A15, hexadecanoyl-CoA; A16a, acetoacetyl-CoA; A16b, acetoacetyl-[acp]; A17, butanoyl-CoA. ii) carboxylates: C1, acetate; C2, 3-oxopropanoate; C3 glycolate; C4, malate; C5, 3-methyl-2-oxobutanoate; C6, 4-methyl-2-oxopentanoate; C7, 3-methyl-2-oxopentanoate; C8, (S)-methylmalonate semialdehyde; C9, L-aspartate; C10, butanoate; C11, 4-aminobutanoate; C12, L-glutamate; C13, succinate semialdehyde; C14, succinate; C15, hexadecanoate; C16, isocitrate; C17, citrate; C18, oxaloacetate; C19, 3-phospho-D-glycerate; C20, acetoacetate; C21, salicylate; C22, 3-oxoadipate; C23, 3,4-dihydroxymandelaldehyde; C24, chorismate; C25, 6-oxohexanoate; C26, 2-oxoglutarate. iii) amino-group containing: N1, histamine; N2, anthranilate; N3, 5-hydroxytryptamine; N4, 2-amino-4-hydroxy-6-(erythro-1,2,3-trihydroxypropyl)dihydropteridinetriphosphate; N5, Nicotinate; N6, 1,4-butanediamine; N7, 2-hydroxyethyl-ThPP.

The highly represented annotations related to metabolic reactions were mapped on a metabolic map, representing most generic biochemical pathways, using the webtool iPATH [108] (Figure 6). It was immediately clear from the patchy appearance that the retrieved genetically associated functions did not seem to converge on certain predefined metabolic pathways like for instance glycolysis or amino acid biosynthesis. However, when the metabolites that were involved in the mapped reactions were inspected, a surprisingly clear picture emerged. These metabolites included various carboxylates (e.g. glutamate, acetate, propionate, butanoate, formate), charged coenzyme A (which is related to fatty acid synthesis or the TCA cycle), (deoxy-)nucleotides, several nitrogen-containing metabolites including various amino-sugars, and the central glycolytic molecules (e.g. pyruvate, phosphoenolpyruvate, 3-phosphoglycerate and 3-phosphoglycerol). Together, the metabolites constitute the essential precursors for the biosynthesis of EPS (i.e. activated (amino-)sugars [109]), lipoprotein and phospholipids (i.e. 3-phosphoglycerol, fatty acids and amino acids [110]), (lipo-)teichoic acids (i.e. polyols, activated sugars, alanine and ribitol or 3-phosphoglycerol [111]), LPS (i.e. activated amino-sugars, activated C6 and C7 sugars, 2-keto-3-deoxyoctonate (kdo) and fatty acids [112]), and peptidoglycan (activated amino-sugars, glutamine, alanine and diaminopimelate or lysine [113]) [114]. Indeed, the essential reactions of the related synthesis routes appeared to be highly represented and conserved. The above findings strongly support a common regulatory role for Sigma-54 in the control of the biosynthesis of the bacterial exterior.

## Discussion

We have applied a coherent comparative sequence-based strategy to search for functional themes that are common to Sigma-54 mediated control. The strategy basically consisted of three semi-independent comparative analyses concerning: i) the taxonomic distribution; ii) the Sigma-54 activator content; and iii) the genomic context of Sigma-54, its activator and the characteristic promoter. In principle, the former and the latter analysis can be used to identify the functional theme that is associated to any bacterial regulator.

One of the main challenges we encountered in the analysis of the genetic context of Sigma-54, its EBP^54^-activators and the Sigma-54 promoters, was the fact that most of the function information that is gathered in larger resources in the public domain is given and viewed in terms of a limited set of established biochemical pathways and/or functional classes. As a result, the recovered genetic associations *per se *did not reveal a single over-represented functional category (using COG categories) or a complete pathway (using KEGG orthologs), in line with earlier observations. However, changing the perspective from isolated categories and pathways to a more integrated (systems) view, and from pathways to compounds, all of a sudden a coherent pattern emerged (see Figure 6). Most of the conserved reactions and transport systems relate directly or indirectly to the biosynthesis of EPS, lipoprotein, (lipo-)teichoic acids, LPS, peptidoglycan and phospholipids, by producing and/or transporting the particular precursors/building blocks. In fact, this common functional theme of being a controller of the synthesis of the bacterial exterior covers very well the diversity within the reported physiological effects of Sigma-54. In the following we will discuss the foundation of this assessment in more detail.

### Considerations based on conservation

A clear relation between the activity of Sigma-54 and the synthesis of flagella and or pili has been established in a wide variety of bacterial species (see e.g. [34,35,49,50,115-118] and reviews by [71,119]). Indeed, this association appears very general considering the strong correlation between the presence of Sigma-54 and cellular motility (Figure 2C). Moreover, a comparison of the presence/absence distribution with the transition-based tree of life advocated by [120,121] was in line with this generalization. Those species that were proposed to have developed before the advent of flagella lack Sigma-54, whereas the majority of species that were proposed to have arisen later have the sigma factor.

The classification of the various phyla based on morphological features (see [63]) as presented in Figure 1, revealed another clear functional association. Sigma-54 is mainly present in diderm organisms that synthesize LPS. Although the association is not absolute, a closer inspection of the diderm species that lack Sigma-54 showed that more than 90% of them are (facultative) endosymbionts. The organisms of the phylum Cyanobacteria constitute a notable exception. However, it has been reported that their LPS is distinct from that of the other LPS-containing diderm species [122]. Thus, a straightforward comparison of the morphology and mobility of species that have Sigma-54 versus species that do not, provides a clear link between the sigma factor and the presence of flagella and the biosynthesis of LPS. In fact it was shown recently, that the assembly of the flagella and the LPS in *Campylobacter jejuni *is an associated process and linked through a single enzyme [123].

We have collected a complete set of EBP^54^s for the analyzed genomes and classified them on basis of domain structure and similarity to experimentally verified activators. The annotation of the main groups of EBP^54^s suggested a clear relation to the biosynthesis of the bacterial exterior and the transport and synthesis of the required precursors. The most dominant activator subgroup was formed by the AtoC-like activators (of type Ia) and was represented in all but one of the analyzed phyla. The main family member AtoC has been associated in the literature with the catabolism of short-chain fatty-acids, the biosynthesis of polyhydroxybutyrate and with the expression of flagellar genes [124].

The common functional theme was also perfectly reflected in the genomic context of the Sigma-54 encoding genes. More specifically, in many Firmicutes the enzymes of the lower branch of glycolysis are genetically associated, thus linking Sigma-54 to the synthesis of 3-phosphoglycerate and phosphoenolpyruvate (PEP). The former compound can easily be converted to 3-phosphoglycerol, whereas PEP is essential to convert UDP-N-acetylglucosamine to UDP-N-acetylmuramic acid during the formation of peptidoglycan [113]. In addition, PEP serves as free energy donor in the transport of (amino-)sugars mediated by the PTS. In most diderms the LPS transport system [102-104] and enzymes involved in synthesis of the building block of the LPS core (2-keto-3-deoxyoctonate, kdo) [105] are genetically associated to the Sigma-54 encoding gene. In fact, it has been proposed that this association represents the ancestral state of all Proteobacteria [125]. The same holds for *yhbJ*, a gene that encodes an important regulator of the glucosamine-6-phosphate synthase and is controlled by Sigma-54 in *E. coli *[101], where glucosamine-6-phosphate is the main amino-sugar precursor in the synthesis of LPS and the bacterial cell wall [112-114]. Interestingly, in the enterobacteria, Sigma-54 also controls the activity of glucosamine-6-phosphate synthase via the expression of the small RNA regulators GlmY and GlmZ [99,100].

### The direct links to central nitrogen and central carbon metabolism

A specific 'nitrogen-PTS' (PTS^Ntr^) and mannose-PTS were also proposed to be part of the ancestral Proteobacterial association [125]. The PTS^Ntr ^is found in Proteobacteria (except for the Epsilon-proteobacteria) [126] and is involved in the integrative regulation of carbon and nitrogen metabolism [37,38,127]. In addition, the PTS^Ntr ^has been connected to the control of biofilm formation in *Vibrio cholerae *[128,129]. Remarkably, the mannose-PTS connection is found in all Firmicutes, either directly like in the Lactobacilli *Lactobacillus plantarum *[23] and *Pediococcus pentosaceus*, or indirectly via the EBP^54^-activators. The latter LevR/CelR-like activators are found in all Sigma-54 containing *Firmicutes *and in some enterobacteria. Their activity is controlled by extracellular carbohydrate and intracellular PEP levels via the PTS [37,59]. The connected PTS systems have been shown to transport cell-wall related carbohydrates [130-133] and belong to the cellobiose and mannose sub-families. It was recently shown by [134] that the mannose-family PTS ManXYZ of *E. coli *plays an important role in the secretion of the glucosamine intermediates from the cytoplasm to the periplasm where the *E. coli *cell wall is assembled.

Regulation by Sigma-54 is clearly linked to central nitrogen metabolism. In a noteworthy attempt to uncover an underlying functional theme for Sigma-54 mediated transcriptional control within *E. coli *[25] concluded that nitrogen assimilation was a major theme and that, considering the widespread distribution of the Sigma-54 system together with the constraints determined by maintenance of promoter integrity, the role of Sigma-54 should be limited to a few physiologically related themes in the various organisms. In fact, considering the composition of the bacterial exterior (listed earlier) and the way the different components are covalently linked, the association with nitrogen assimilation is rather logical. The important precursors of the biosynthesis of the molecules of the exterior are (N-acetyl-)glucosamine, Glu, Lys, PEP and various fatty acids (i.e. carboxylates). Most other precursors can be synthesized out of these. For instance, Glu and PEP can be converted to oxaloacetate and Ala [112], and Lys can be interconverted to diaminopimelate (dap) [135]. In the last case, the biosynthesis route starts at aspartate, the transport of which is controlled by the Sigma-54 dependent two-component system DctBD in many species [136,137]. In addition, many of the moieties are finally covalently linked through peptide bonds, which involve an amino- and a carboxyl-group. Therefore, in order to control the direction and scale of the metabolic fluxes related to the biosynthesis of the different exterior structures the bacterium should control the extent of nitrogen assimilation and the production of the central metabolites Glu and PEP. Nevertheless, the way in which the control is exerted may vary between species. For instance, Sigma-54 is controlling the intracellular Glu levels via glutamate dehydrogenase in the monoderm *B. subtilis *[138], whereas it acts on glutamine synthetase in the diderm *E. coli *(see [25]).

## Conclusion

We have established a clear connection between Sigma-54 and the make-up of the bacterial exterior. The sigma factor exerts its control directly by regulating the expression of the genes involved in the transport and biosynthesis of the main precursors. In some cases, the control is indirect and mediated via an additional regulator like in the case of PTS^Ntr ^[38] or via another sigma factor like Sigma-S in Borrelia [46] or Sigma-32 in enterobacteria [25]. However, experimental evidence for the latter connection has not yet been reported. In contrast to the situation in Borrelia, in *E. coli*, Sigma-54 and Sigma-S have been predicted to act in an antagonistic way [139]. Of course, the control exerted by Sigma-54 should not be viewed as an isolated process or acting at the level of transcription alone. Regarding the latter, there seems to be a connection to control at the level of translation activity (e.g. ribosome hibernation in stationary phase [106]). Regarding the former, because Sigma-54 mediated control will affect various fluxes involving central metabolites, other global regulatory factors bear upon its activity, such as the DNA-bending proteins IHF and CRP, the alarmone ppGpp and the RNA-polymerase targeting protein DskA (reviewed in [18]).

Although the association with the exterior constitutes the evolutionary conserved (i.e. pan-bacterial) functional basis of Sigma-54 mediated control, the extent to which the related processes are controlled will very much depend on the species, as is obvious from the variation in the number of Sigma-54 activators. In addition, other processes might have become linked too because they feed on the same metabolic intermediates. Vice versa, such connections might also have been lost and there are several examples of that. These include the activators DhaR, TyrR and VpsR, which are related to the regulation of the uptake and phosphorylation of dihydroxyacetone [28], of the biosynthesis of aromatic amino acids biosynthesis [27], and of the biosynthesis of a particular EPS in Vibrio cholerae [77], respectively. These activators clearly descended from active Sigma-54 dependent EBP^54^s but now carry a small deletion that has made them Sigma-54 independent.

The regulatory connection to the bacterial exterior explains all of the reported physiological variability related to Sigma-54 very well. For instance, the impact on osmotolerance that was observed for *Listeria monocytogenes *upon deletion of Sigma-54 [39] could very well be related to its role in petidoglycan synthesis. In fact, in *E. coli *turgor pressure is controlled through potassium ion transport via the *kdp *system, a system which in turn is controlled via the PTS^Ntr ^and thus by Sigma-54 [140]. The reported changes in virulence can also be explained perfectly through changes in the LPS composition. In addition, alterations in the motility and the presence of flagella will directly affect the invasive power, as observed for *Borrelia burgdorferi *[47], and also the swarming behavior on surfaces [141]. Related to that, adaptations of the bacterial exterior mediated by Sigma-54 will clearly have to affect the formation and the properties of bacterial biofilms. And this is precisely what has been observed for many species [142]. Sigma-54 and the associated activators thus represent potentially highly effective targets in the areas of food safety and health as changes in the bacterial exterior induce the establishment and affect the stability of deleterious bacterial populations.

## Methods

### External Data and Tools

Genome sequence and annotation information was obtained from NCBI [143]. For all species with a sequenced genome that was published before November 2009, the taxonomic attributes and physiological data were derived from the GOLD database [60]. Other function annotation of genes was automatically collected on basis of gi-IDs from the Uniprot database (PFAM, COG, GO and Interpro) [144] and the KEGG database (Kegg ortholog, pathway, linked reactions and compounds) [145]. Sequence similarity searches were performed using BlastP or tBlastN [146], Hidden Markov Models (implemented according to [147]), or using Similar Motif Search (see below). The latest version of ClustalX [148] was used for multiple sequence alignments and for the generation of Neighbor Joining (NJ-) trees (bootstrapped and corrected for multiple substitutions). NJ-trees were visualized and organized using LOFT [149] or Dendroscope [150]. Frequency representations of aligned sequences were created with Weblogo [151]. Wordles [152] were employed to compare the frequency of annotations. The tool iPATH [108] was used to visualize the metabolic context of recovered sets of annotations. All relevant data has been made publicly available at http://www.cmbi.ru.nl/bamics/supplementary/Franckeetal_2011_Sigma54theme.

### Similar Motif Scoring (SMS)

The identification of stretches of DNA, RNA or protein sequence with a certain function relies on knowledge of other sequences carrying that particular function and a scoring method to characterize the similarity between the target and the query. In general, sequence comparison algorithms evaluate the statistical relevance of the overlap between a target and a given query. Although such an approach is very powerful for larger sequences, it is less discriminative for smaller sequences, like transcription factor binding sites. Therefore, much effort has been put in the development of advanced scoring methods in the field of DNA-binding site identification [153,154]. Nevertheless, ultimately the current tools provide scores and associated rankings that reflect probability rather than similarity.

Most scoring methods that rely on a known aligned set of input sequences create a position weight matrix (i.e. motif) [56,155,156]. Considering the fact that the number of input sequences is normally limited, a proper sampling of the query sequence space is not provided and thus a probabilistic scoring by default will be skewed. In addition, most methods have to introduce artificial scores in case a specific nucleotide is fully conserved or completely absent at a certain position within the input set [157]. Remarkably, a potential solution to both problems has implicitly been provided by many researchers who have compared binding-site predictions with experimentally observed changes in transcript levels. One of the most common practices to reconcile prediction with experiment is to minimize the number of differences between the target and the query (or the 'consensus'). In fact, this criterion can be captured in a straightforward scoring using only the position frequency matrix:

Given any number *m *(≥ 1) of input sequences of size *i*, the nucleotide frequency *f*_N(j) _(where N ∈ A, C, T, G; and frequency is in terms of fraction) at every position *j *= 1 to *i *can be used directly to provide all target sequences of size *i *with a score by adding up the input-based frequencies that relate to the nucleotide composition of the target. Division of the score by the length of the sequence *i *results in a relative 'similarity' score that can range from 0 to 1.

In case the input sequences are representative for high-affinity sites, the ranking of target sequences according to score should approximately correspond to a ranking based on affinity. In addition, the degeneracy of the input motif can directly be deduced from the scores of the individual input sequences and their deviation from 1.

The simple scoring method was implemented in a Similar Motif Search web-tool within the FG-Web framework (van Hijum unpublished, https://trac.nbic.nl/fgweb/) and the effectiveness of the tool was compared to MAST [158] and Hidden Markov Models [147] using the well-studied CcpA [159] and Spo0A [160] regulons in *B. subtilis *as a test case. These test data-sets were chosen because they relate to global regulators (most difficult to predict regulons) and they were experimentally validated. The test indicated that our SMS method is at least as good to identify putative regulatory elements on basis of known input motifs as the commonly used tool MAST [158] (see additional file 8). In fact, within this 'new' similarity scoring no assumptions have to be made, other than that the given input set represents the sequence one is looking for. Moreover, the SMS method was easily adapted to search specific amino acid motifs in sets of proteins and was successfully used to separate true EBP^54^s from homologs not related to Sigma-54.

### Promoter identification

The 85 experimentally verified promoters collected by [14] were used to formulate an initial Sigma-54 promoter sequence motif. The promoter motif was then reduced by smoothing those frequencies that appeared to be rather random, to arrive at a frequency representation of the Sigma-54 promoter as given in Figure 4C. Although the consensus promoter was valid for species that have a single copy of Sigma-54, it should not necessarily be the case for species that carry multiple copies of the sigma factor. These species include *Bradyrhizobium japonicum *[66], *Rhizobium etli *[67], *Rhodobacter sphaeroides *[69], *Xanthomonas campestris *[68], and a few others. It was reported that the promoters related to the paralogs RpoN1 and RpoN2 in *Rhodobacter sphaeroides *vary only at nucleotide position -11 [70]. Similarly, for *Bradyrhizobium japonicum *two promoter consensus sequences were identified that differed only at position -12 [161]. Considering the fact that the observed natural promoter variability involves single nucleotide substitutions, we assume that our promoter predictions are also relevant for the species with multiple Sigma-54 copies. Indeed, for *Rhodobacter sphaeroides *and *Bradyrhizobium japonicum *the high-ranking putative promoters included representatives of both reported promoters (not shown).

### Identification of Sigma-54 and the related EBPs

The sequences of the experimentally characterized Sigma-54 of *E. coli *and *B. subtilis *and 34 experimentally characterized EBP^54^s (see additional file 3 for sequences and references) were used to identify homologs in all sequenced prokaryotic species (data from NCBI non-redundant protein database as of 1^st ^November 2009). In the case of Sigma-54 no selection was needed. In the case of the EBPs a selection of true positives was performed in three steps. First, all hits were filtered on basis of a maximum product of all 34 e-values (< 1e^-5^) and then on basis of a maximum sum of all 34 e-values (< 1). To remove remaining false positives the presence of the characteristic 'GAFTGA' amino acid sequence that is essential for the interaction between the activator and the sigma factor [74] was analyzed (as discussed in the main text). A comparison between the number of genes encoding a true Sigma-54 activator as identified by us and the number of genes reported in the Uniprot database implies that the Uniprot database contains a relatively large number of false-positive identifications. For species lacking Sigma-54, the Uniprot data suggests that 20% has an EBP^54^. Moreover, for species that have Sigma-54 the total number of EBP^54^s listed within the Uniprot database is only correct in 42% of the cases (see additional file 1). Many of the false identifications relate to the lack of filtering on basis of the integrity of the 'GAFTGA' element. Unfortunately, these false identifications extend into the literature. For instance *E. coli *DhaR [25], *V. cholerae *VpsR [77] and *B. subtilis *YplP [24], have been mistakenly ranked as Sigma-54 activators, whereas they clearly lack the 'GAFTGA' element (additional file 3).

### Implementation of Context analysis

The gi-IDs of the putative Sigma-54 and EBP^54 ^proteins and the genomic position of the Sigma-54 related promoters were used to collect annotation information related to neighboring genes from the reference databases (i.e. NCBI, Uniprot, KEGG and GOLD). In case of the promoters, only those that were correctly oriented and spaced (-50 to 300 nucleotides from translation start) and were within the top 15 ranked identified promoters, were used. The retrieval of annotation information associated to the genomic context was encoded in Python and the code was then converted into a local 'context connector' web-tool within the FG-Web framework (van Hijum unpublished, https://trac.nbic.nl/fgweb/). The tool allowed the definition of constraints related to: i) the number of genes neighboring the query gene that should be taken into account; or ii) the organization of those genes, i.e. in 'operons' or 'divergons' (defined using a distance criterion of maximally 300 nucleotides between genes).

## List of abbreviations

COG: Cluster of Orthologous Genes; CW: Cell Wall; dap: diaminopimelate; EBP: Enhancer Binding Protein; EC: Enzyme Code; ECF sigma factor: ExtraCytoplasmic Function sigma factor; EPS: ExoPolySaccharide; GO: Gene Ontology; HMM: Hidden Markov Model; HTH: Helix Turn Helix; ID: Identifier; kdo: 2-keto-3-deoxyoctonate; LPS: LipoPolySaccharide; NJ: Neighbor Joining; OM: Outer Membrane; PEP: PhosphoEnolPyruvate; PTS: PhosphoTransferase System; SMS: Similar Motif Search; TCA: TriCarboxylic Acid; TCS: Two Component System.

## Authors' contributions

CF conceived, designed and coordinated the study, carried out motif and functional analyses and drafted and revised the manuscript. TGK conceived and designed the motif search algorithm and carried out motif analysis and helped revising the manuscript; YH implemented the motif search algorithm and validated the algorithm; LO conceived and designed the tool to collect context information and helped revising the manuscript; VS implemented the tool to collect context information and validated the tool; RM and RJS coordinated the study and helped drafting and revising the manuscript. All authors read and approved the final manuscript.

## Supplementary Material

Additional file 1 **Presence-Absence analysis for genes encoding Sigma-54 and the related Enhancers**. The file contains: sheet 1 with the results of the presence analysis of Sigma-54 and the related Enhancers in all bacterial genomes published before November 2009 listed in the GOLD database and with NCBI NC-code; and sheet 2, which provides a summary of the presence and absence in relation to genome size. Click here for file

Additional file 2 **All collected Sigma-54 related EBPs with annotation**. The file contains: sheet 1, which gives the complete list of putative Sigma-54 related EBPs for the studied genomes (given in additional file 1) in order of product of BLAST e-values related to experimentally verified EBPs (given in additional file 3); sheet 2, which provides the putative annotations; and sheet 3, which gives the annotation summary. Click here for file

Additional file 3 **List of experimentally validated EBPs and a multiple sequence alignment of the 'GAFTGA' region**. The file contains: sheet 1, which provides the list of EBPs that was used to search EBP homologs with BLASTP; sheet 2, which gives the list of EBPs that was used to annotate EBP homologs with reference to relevant experimental data in second column; sheet 3, which shows a snapshot of the multiple sequence alignment of the EBPs from sheet two in the GAFTGA region; sheet 4, which gives the list of EBPs with a defective GAFTGA sequence; and sheet 5, providing a summary of the related numbers. Click here for file

Additional file 4 **Promoter predictions for model organaisms**. The file contains: the promoter predictions for *Bacillus subtilis *(sheet 1), *Escherichia coli *(sheet 2), *Lactobacillus plantarum *(sheet 3) and *Pseudomonas putida *(sheet 4); and a summary of the predictions (sheet 5). Click here for file

Additional file 5 **Representation of the gene annotations in the context of the Sigma-54 encoding genes**. The file contains: sheet 1, giving the annotations present in the context of the Sigma-54 encoding genes in all species (10 genes upstream and 10 genes downstream); and a summary sheet 2. Click here for file

Additional file 6 **Representation of the gene annotations in the context of the EBP encoding genes**. The file contains: sheet 1, giving the annotations present in the context of the operons and divergons that contain a gene encoding an EBP (max 10 genes upstream and 10 genes downstream and max 300 nucleotides intergenic distance); and a summary sheet 2. Click here for file

Additional file 7 **Representation of the gene annotations in the context of the Sigma-54 promoters**. The file contains: sheet 1, giving the annotations present in the genes and operons that follow a correctly spaced and oriented Sigma-54 promoter (member of top 15 ranked hits)(1 gene upstream and max 10 genes downstream); and a summary sheet 2. Click here for file

Additional file 8 **Validation of the Similar Motif Search Procedure**. The file contains: sheets 1, 2 and 3, with the outcome of a comparison between SMS and MAST in the identification of CcpA and Spo0A regulon members in *Bacillus subtilis*; and sheet 4 with a summary of the comparison. Click here for file
